# Accuracy of magnetic resonance imaging in detecting lumbo-sacral nerve root compromise: a systematic literature review

**DOI:** 10.1186/s12891-016-1236-z

**Published:** 2016-09-06

**Authors:** Nassib Tawa, Anthea Rhoda, Ina Diener

**Affiliations:** 1 Department of Rehabilitative Sciences, College of Health Sciences, Jomo Kenyatta University of Agriculture & Technology, P.O. Box 62 000 00200, Nairobi, Kenya; 2 Department of Physiotherapy, Faculty of Community and Health Sciences, University of the Western Cape, Private Bag X 17, Bellville, 7535 Republic of South Africa

**Keywords:** Lumbo-sacral radiculopathy, Accuracy, MRI, Diagnosis

## Abstract

**Background:**

MRI is considered to be the diagnostic tool of choice in diagnosing nerve root compromise among patients presenting with clinical suspicion of lumbo-sacral radiculopathy. There exists controversy among researchers and clinicians regarding the diagnostic utility and accuracy of MRI in detecting nerve root compromise and radiculopathy. This review evaluated 4 primary diagnostic accuracy studies that specifically assessed the accuracy of MRI in detecting nerve root compromise, as established in the current literature.

**Methods:**

Eight electronic data bases were searched for relevant articles from inception until January 2014. All primary diagnostic studies which investigated the accuracy of MRI in diagnosing nerve root compromise among patients with low back and referred leg symptoms were screened for inclusion. Qualifying studies were retrieved and independently assessed for methodological quality using the ‘Quality Assessment of Diagnostic tests Accuracy Studies’ criteria.

**Results:**

Four studies qualified for inclusion in this review. The sensitivity of MRI in detecting lumbar nerve root compromise was very low at 0.25 (95 % CI) while the specificity was relatively high at 0.92 (95 % CI).

**Conclusions:**

There is lack of sufficient high quality scientific evidence in support or against the use of MRI in diagnosing nerve root compression and radiculopathy. Therefore, clinicians should always correlate the findings of MRI with the patients’ medical history and clinical presentation in clinical decision making.

## Background

MRI is frequently used in examining patients with Lumbo-Sacral Radiculopathy [1, 2]. Access to advanced imaging technology is proposed to improve diagnostic accuracy and facilitate effective treatment for better health outcomes [3]. However, in some clinical instances, the relationship between MRI-visible anatomical abnormalities, clinical history and patients’ treatment outcomes remain controversial [4, 5]. Similarly, there are documented reports of high prevalence of MRI-visible lumbar spine abnormalities in asymptomatic subjects [6].

Currently, in the field of musculo-skeletal medicine, there have been reports of over-utilisation and over-dependency on imaging, which has been attributed to among other reasons technological advances, availability of medical imaging, clinicians’ uncertainty and patients’ expectations [7]. These may all result from clinicians’ attempt to address the delicate balance between not missing a treatable pathology and avoiding unnecessary investigation which may increase patients’ fears about their condition [8].

MRI examination of the lumbo-sacral spine is proposed to provide detailed anatomic assessment of the spine, however, it has a high potential of identifying incidental findings which are morphologically abnormal but not responsible for, or even related to, patients’ symptoms [9]. Lumbo-sacral MRI findings may sometimes be irrelevant in clinical decision making and ultimate treatment outcomes [5]. Such findings may influence further investigations, unnecessary treatment options, increased cost of care and possibly poor outcomes [5, 9]. MRI of the lumbo-sacral spine has been proven to be able to detect alterations in both the anatomy (disc herniations and spinal canal stenosis) and tissue properties (disc desiccation and reactive marrow changes), which then need to be considered within a clinical context [4]. Other characteristics investigated by MRI include disc contour abnormalities (bulge and herniations), and degenerative changes of the inter-vertebral discs, bone marrow, neuro-foramina, spinal canal and facet joints [5]. The diagnostic utility of MRI in assessing normal lumbar anatomy, internal disc chemistry and architecture, features of lumbar spine degeneration, and in diagnosing herniated lumbar discs have been well documented [4, 5, 7]. However, it’s accuracy in detecting nerve root compromise remains questionable as evident by conflicting reports by Bertilson et al. [1] that MRI is insensitive and Kreiner et al. [2] that MRI is sensitive and thus recommended for diagnosing LSR.

Abnormal lumbo-sacral imaging findings in patients with LSR are in some instances coincidental, hence the need to correlate imaging findings with the patient’s clinical picture [4, 5, 10]. This shortcoming, on the likelihood of false positive findings on MRI, coupled with high economic cost of radiological imaging, and the surgical interventions they may trigger, has invoked consistent criticism among authorities in the fields of neurology and musculo-skeletal heath care as indicated earlier in the American Agency for Health Care Policy and Research (AHCPR) recommendations by Bigos et al. [11] and recently by Weiner and Patel [4]; Lysdahl and Hofmann [8].

These authors recommended that clinicians should correctly apply and understand the limitations of MRI examination in the assessment of patients suspected with LSR.

The current review of the literature therefore sought to establish the sensitivity and specificity of MRI in detecting lumbo-sacral nerve root involvement among patients with low back and referred leg pain.

The aim of this review was to determine the accuracy of MRI in detecting lumbo-sacral nerve root compromise, as reported in the literature. The diagnostic accuracy measurements which were established in this review included validity, reliability, sensitivity and specificity.

## Methods

This review was conducted using the diagnostic tests accuracy (DTA) protocol of the Cochrane Collaboration [12]. The authors adopted the Cochrane DTA format since it helps readers to find the results of reviews quickly and to assess the validity, applicability and implications of those results. It also guides review authors to report their work explicitly and concisely.

### Search strategy

**Fig. 1:**
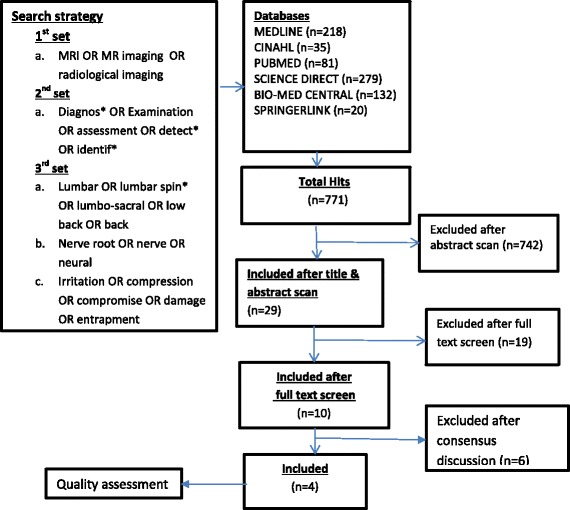
Search history

The reviewers developed and conducted a structured literature search from May 2012 up to February 2014 to identify relevant studies in various electronic databases including MEDLINE, CINAHL, Biomed Central, Science Direct, Springerlink, Google scholar, Pubmed, and Embase. No publication date limitation was imposed, thus all databases were searched since inception up to February 2014. The search was performed by the first reviewer (NT), followed by reference tracing of potentially relevant articles complemented by hand searching of field- and topic-relevant journals including reference lists of potentially relevant articles. The search strategy as illustrated in Fig. 1 incorporated synonyms, related terms, variant spelling, truncation and Boolean operators using the following MeSH terms;

- *1*^*st*^*set*
  - *MRI OR MR imaging OR radiological imaging*
- *2*^*nd*^*set*
  - Diagnos* OR Examination OR assessment OR detect* OR identif*
- *3*^*rd*^*set*
  - Lumbar OR lumbar spin* OR lumbo-sacral OR low back OR back
  - *Nerve root OR nerve OR neural*
  - *Irritation OR compression OR compromise OR damage OR entrapment*

### Study selection criteria

Selection of studies for the purposes of this review was independently performed by two reviewers (NT and ID) using the PICO analysis (Booth and Fry-Smith 2003) and disagreements were resolved through discussion and the opinion of a third reviewer (AR). The studies were pre-screened according to:

- Participants: For studies to be included in this review, the sample must have been patients aged 18 years and older presenting with low back and referred leg pain or back-related leg pain, and not previously diagnosed with specific serious pathologies like fractures, tumors and infections of the lumbar/sacral spine causing low back and/or referred leg symptoms.
- Index tests: This review only included studies which examined any aspect of MRI parameters relevant to nerve root compromise using screening or limited protocol MRI, routine full protocol MRI, or diffusion-weighted imaging (DWI). The parameters which are relevant to nerve root compression are significant protrusion of inter-vertebral disc material (nucleus pulposus) and spinal stenosis, compromising nerve roots. The reviewers thought it was necessary to only focus on MRI parameters which are specific to nerve root compromise so as to conform to the ISAP definition of radiculopathy.
- Target Condition: This review targeted primary diagnostic studies whose main aim was to detect LSR due to nerve root compromise using MRI. Studies whose target condition was other specific causes of LSR (like tumors or infections of the spine) other than nerve root compromise were excluded.
- Outcomes: Reference standards: The reviewers included diagnostic studies which compared the accuracy of MRI against acceptable comparators like clinical neurological examination (testing of sensory, motor, tendon reflex and neuro-dynamic properties), pain drawing, fluoroscopic radiculography, electro-diagnostics (EMG), lumbar medial nerve blockade, plain Computed Tomography (CT), CT myelography and intra-operative findings.

Table 1 illustrates the PICO analysis of the studies which qualified for inclusion in this review.

**Table 1:** PICO analysis of included studies

Author (year)	Patients description	Index test	Comparison	Outcome
Hasankhani & Omidi-Kashani 2013 [17]	152 patients	MRI	CNE & eclectro-diagnostics	MRI showed a high + likelihood ratio for nerve root involvement indicating that it is a better modality to confirm radiculopathy.
	15 years and older			
	Radicular low back pain			
Eguchi (2011) [16]	18 years and older	Diffusion-Weighted Imaging (DWI)	Routine MRI	Mean ADC values were significantly greater in the compressed DRG and distal spinal nerves than in intact nerves.
	10 patients with			
	Mono-radicular symptoms			
Bertilson (2010) [1]	18 and older	MRI	CNE and simplified pain drawing	Structured physical examination (including CNE), and pain drawing showed more sensitivity than MRI for nerve involvement.
	61 patients with long-standing nerve root symptoms			
Thornbury et al. (1993)	18 and older	MRI	Plain CT and CT myelography	No statistically significant difference in the diagnostic accuracy of MRI, plain CT and CT myelography in the diagnosis of nerve root compression caused by HNP.
	95 patients with acute low back and radicular pain			

### Quality assessment

Two reviewers (NT and ID) independently assessed the quality of the four included studies using the Quality assessment of Diagnostic Accuracy Studies (QUADAS) criteria and scoring disagreements between the two reviewers were resolved by a discussion until a consensus was reached.

Each of the included studies was separately assessed for each of the 12 items. Studies were scored as ‘positive’ (+), when the described methodology was of good quality according to the guidelines of the QUADAS criteria, as ‘negative’ (−), when the described methodology was not of acceptable quality, and ‘not sure’ (?), when the methodology was inadequately described.

### Data extraction

The first review author (NT) independently extracted data from the original studies using a self-developed data sheet. Data extraction covered participants (total number, age, gender, clinical characteristics, clinical setting and recruitment period), examiners (number, expertise and experience) and assessment procedure/tools.

### Statistical analysis and data synthesis

The reviewers extracted, or where unavailable re-calculated the common parameters of diagnostic test accuracy including; sensitivity, specificity, positive likelihood ratio (+LR), negative likelihood ratio (−LR) and diagnostic odds ratios (DOR). Also, true positive, false positive, true negative and false negatives of each investigated index test is presented. A meta-analysis was not conducted given the minimal numbers of included studies in this review. Where necessary, and in cases where raw data were incomplete, a 2 × 2 contingency table was used to re-calculate the diagnostic accuracy values.

### Data analysis

The reviewers extracted, and where unavailable re-calculated the common parameters of diagnostic test accuracy including; sensitivity, specificity, positive likelihood ratio (+LR) and negative likelihood ratio (−LR). Also, true positive, false positive, true negative and false negatives values of all investigated index tests were recorded.

However, as suggested by Pepe et al. [13], diagnostic odds ratios were not calculated in this review due to its limitations in gauging the performance of a diagnostic marker. A meta-analysis was also not conducted given the minimal numbers of included studies in this review. In order to establish the level of agreement between the two observers, a statistical technique was applied by using un-weighted Cohen’s Kappa test with 2×2 cross-tabulation in SPSS computer software version 21. The inter-observer agreement between the two reviewers was assessed for each QUADAS item for all included studies.

The QUADAS criteria which were developed by Whiting et al. [14] are a methodological checklist which is used to assess the quality and design of primary diagnostic studies. The checklist comprises of questions on the spectrum of the participants who were included in the study, the inclusion criteria, description of target condition, index test and reference standard and interpretation of test results. Kappa (k) values and P- values were considered as indicators in determining the statistical significance of the observed agreement. The inter-observer agreement was considered poor if k ≤ 0, slight k ≤ 2, fair k ≤ 4, moderate k ≤ 6, good k ≤ 8 and perfect k > 8 [15]. Scoring disagreements were resolved through a consensus discussion between the two reviewers (NT and AR) with the arbitration of the third reviewer (ID) until agreement on all items for all the studies was reached. Where necessary, and in cases where raw data were incomplete, a 2 × 2 contingency table was used to re-calculate the diagnostic accuracy values.

## Results

### Search results

The search on relevant databases yielded a total of 769 articles which were generated by the first hit of the key search terms and the MeSH terms. After removal of duplicates, a screening procedure was done by scanning the abstracts and titles of the search results, 27 articles were pre-qualified as suitable for PICO analysis. Out of the 27 articles, 12 were selected from those that were generated by the entry of the key search terms while 15 were selected from the output of the MeSH terms.

Full screening of the 27 articles was independently done by two reviewers (NT & ID) using a PICO analysis and nineteen studies were further excluded.

A discussion was held between the two reviewers (NT and ID) with adjudication by the third reviewer (AR) regarding the specific objectives of the eight remaining studies and a further four were excluded because their primary objective was simply to assess the accuracy of MRI in detecting disc herniation and not nerve root compromise. Only four studies were finally qualified for inclusion in this review. Three of the studies (Bertilson et al. [1]; Eguchi et al. [16]; Hasankhani and Omidi-Kashani [17]) are relatively recent and were done in Sweden, Japan and Iran respectively. The fourth and older study (Thornbury et al. 1993) was done in USA. All four studies assessed the accuracy of MRI in detecting lumbar nerve root compromise among patients who presented with signs and symptoms consistent with LSR. Three studies (Thornbury et al. 1993; Bertilson et al. 2010 [1]; Hasankhani and Omidi-Kashani [17]) were cohort studies and used electro-diagnostics, clinical examination and simplified pain drawing and CT myelography as reference standards while the Eguchi et al. [16] was a case control study which used healthy volunteers as controls according to findings on an ordinary MRI. Figure 1 below illustrates the search process.

### QUADAS scores of reviewed studies

The final QUADAS scores for the four included studies across all QUADAS items are presented horizontally in Table 2 and this was calculated as a percentage of the sum of all positive scores divided by the total number of QUADAS items (12). Therefore, the quality scores were 50 %, 58 % and 75 % for Hasankhani and Omidi-Kashani [17]; Eguchi et al. [16] and Bertilson et al. [1] respectively. All studies did not fulfil criteria items 4 and 11, meaning there was no clear explanation regarding the delay between MRI examination and application of the reference standard which might have caused disease progression or recovery bias and also the authors in all four studies did not report un-interpretable results.

**Table 2:** Methodological quality assessment of reviewed studies using QUADAS criteria

Author (year)	Criteria number												
	1	2	3	4	5	6	7	8	9	10	11	12	%
Hasankhani & Omidi-Kashani 2013 [17]	+	+	+	?	+	+	-	-	?	?	+	-	50
Eguchi, et al. (2011) [16]	+	?	+	-	+	+	+	+	-	+	-	-	58
Bertilson et al. (2010) [1]	+	+	?	-	+	+	+	+	+	+	-	+	75
Thornbury et al., 1993	+	+	?	-	+	+	-	+	?	-	-	+	50
% of maximum	100	75	50	0	100	100	50	75	25	50	25	50	

The sensitivity and specificity of MRI in detecting lumbar nerve root compromise were extracted from included studies. Diffusion-weighted Imaging (DWI) which uses similar principles and techniques like routine MRI was used in the other reviewed study. It is a recent technological advancement in the field of medical imaging which offers an alternative means to assess the morphology of suspected nerve roots through measurement of the apparent diffusion coefficient (ADC) (Eguchi et al. [16]). The two studies which were reviewed gave a satisfactory and elaborate explanation of the imaging equipment and process.

In the Eguchi et al. [16] study, a 1.5-Tesla scanner (Achieva 1.5 T Nova Dual; Philips Medical Systems, Japan) was used for image acquisition. During the examination process, subjects were scanned in supine position using a sense XL Torso coil, and diffusion-weighted imaging (DWI) was performed with a background body signal suppression and short T1 inversion recovery-echo planar imaging sequence. The results indicated that the mean apparent Diffusion Coefficient (ADC) was greater at compressed DRG and distal spinal nerves than in the controls. In this reviewed study, MRI could detect compromises at and below site of compression. In the Bertilson et al. [1] cohort study, a 1.0 Tesla scanner (Philips Intera) was used for image acquisition. Patients were positioned in supine and a phased array spinal coil was used to produce sagittal and axial T1 and T2 spin and turbo spin echo sequences (slice thickness 3 mm, inter-slice gap 0.3 mm, fields of view 25 cm for sagittal and 16 cm for axial images). The reported outcome was that MRI-visible nerve involvement at any location and segment was less compared to the reference standard of physical examination findings.

The sensitivity of MRI in detecting lumbar nerve root compromise was very low at 0.25 (95 % CI) while the specificity, which is the probability of getting a negative MRI test result on a patient with negative findings for nerve root compromise by physical examination, was relatively high at 0.92 (95 % CI).

## Discussion

This review aimed at establishing the accuracy of MRI in diagnosing lumbo-sacral nerve root compromise as one of the causes of radiculopathy, and not detection of disc herniation and sciatica. The main finding of this review is that there is not sufficient high quality evidence for or against the use of MRI in diagnosing Lumbo-Sacral nerve root compromise and Radiculopathy. Most previous primary diagnostic studies and reviews focused on assessing the accuracy of MRI in detecting lumbar disc herniation and sciatica and not radiculopathy. This could be attributed to the misinterpretation of the diagnostic accuracy of MRI since other bio-mechanical causes may also lead to nerve root compromise and radiculopathy [5, 18].

Similarly, it has been reported that MRI cannot detect far-out possible extra-foraminal causes of radiculopathy and that MRI-visible nerve root compromise does not necessarily mean radiculopathy, and vice vasa [5, 19].

Therefore, the use of MRI by clinicians in the diagnosis of LSR could only be attributed to various factors ranging from availability of imaging equipment to mere personal preference by clinicians. Because, on the contrary, very little high quality scientific research has been done to investigate the accuracy of MRI in detecting nerve root compromise and radiculopathy.

Also, the results of the Bertilson et al. study [1] indicate that MRI is rather insensitive in detecting nerve root compromise compared to clinical examination. This runs a risk of registering false negatives contrary to a long held notion that MRI [1]. Electro-diagnosis showed a high positive correlation with MRI in detecting radiculopathy [17] and is a good investigation to confirm the condition especially when the cause is non-discogenic and extra-spinal. The Thornbury et al. study was conducted over two decades ago when imaging and electro-diagnostic technology was not well advanced, hence the findings should be considered with caution.

Recent studies [16, 20, 21] indicate that the future of imaging has a great potential of improving the diagnostic utility through advanced imaging techniques like Diffusion Weighted Imaging (DWI), Diffusion Tensor Imaging (DTI) and Magnetic Resonance Neurography (MR Neurography).

## Conclusions

MRI is regularly used by clinicians in making a decision of whether to treat a patient conservatively using physiotherapy, rehabilitation and pain medication or consider surgical intervention. There is a documented trend on increasing excessive utilisation and over-dependency on MRI in assessing lumbar spine disorders among clinicians. Therefore, based on the findings of this review, the lack of sufficient high quality scientific evidence in support or against the use of MRI, the on-going debate among experts regarding the cost, diagnostic utility and accuracy of MRI in diagnosing nerve root compression and radiculopathy, clinicians should always correlate the findings of MRI with the patients’ medical history and clinical presentation in clinical decision making.

## Abbreviations

MRI: Magnetic resonance imaging
QUADAS: Qualify assessment of diagnostic accuracy studies
PICO: Patient, indicator, comparison, outcome
IVD: Inter-vertebral disc
CT scan: Computed tomograpy scan
EMG: Electro-myography
LBP: Low back pain

## References

[1] Bertilson BC, Brosjo E, Strender L (2010). Assessment of nerve involvement in the lumbar spine: agreement between magnetic resonance imaging, physical examination and pain drawing findings. BMC Musculoskelet Disord.

[2] Kreiner (2014). An evidence-based guideline for the diagnosis and treatment of lumbar disc herniation with radiculopathy. Spine J.

[3] Hilal K, Sajjad Z, Sayani R, Khan D (2013). Utility of limited protocol magnetic resonance imaging lumbar spine for nerve root compression in a developing country, Is it accurate and cost effective?. Asian Spine J.

[4] Weiner BK, Patel R (2008). The accuracy of MRI in the detection of lumbar disc containment. J Orthop Surg Res.

[5] Carrino JA, Lurie JD, Tosteson ANA, Tosteson TD, Carragee EJ, Kaiser J, Grove MR, Blood E, Pearson LH, Weinstein JN, Herzog R (2009). Lumbar spine: reliability of MR imaging findings. Radiology.

[6] Jensen MC, Brant-Zawadzki MN, Obuchowski N, Modic MT, Malkasian D, Ross JS (1994). Magnetic resonance imaging of the lumbar spine in people without back pain. N Engl J Med.

[7] Jarvik JG, Deyo RA (2002). Diagnostic evaluation of low back pain with emphasis on imaging. Ann Intern Med.

[8] Lysdahl KB, Hofmann BM (2009). What causes increasing and unnecessary use of radiological investigations? a survey of radiologists’ perceptions. BMC Health Serv Res.

[9] Bajpai P, Saini S, Singh R (2013). Clinical correlation of magnetic resonance imaging with symptom complex in prolapsed intervertebral disc disease. Web Publication.

[10] Hoogendoorn WE, van Poppel MNM, Bongers PM, Koes BW, Bouter LM (2000). Systemic review of psychosocial factors at work and private life as risk factors for back pain. Spine.

[11] Bigos S, Bowyer O, Braen G (1994). Acute low back problems in adults. Clinical practice guideline no. 14. AHCPR publication no. 95–0642.

[12] Cochrane Style Guide Working Group (2009). Cochrane style guide basics.

[13] Pepe MS, Janes H, Longton G, Leisenring W, Newcomb P (2004). Limitations of the odds ratio in gauging the performance of a diagnostic, prognostic, or screening marker. Am J Epidemiol.

[14] Whiting P, Rutjes AW, Reitsma JB, Boyssut PM, Kleijnen J (2004). The development of QUADAS: A tool for the quality assessment of studies of diagnostic accuracy included in systematic reviews. BMC Med Res.

[15] Landis JR, Koch GG (1977). The measurement of observer agreement for categorical data. Biometrics.

[16] Eguchi, et al. Difusion-weighted magnetic resonance imaging of symptomatic nerve root of patients with lumbar disc herniation. Diagn Neurol. 2011;53:633–41. 10.1007/s00234-010-0801-7 21080158

[17] Hasankhani EG and Omidi-Kashani F. Magnetic resonance imaging versus electrophysiologic tests in clinical diagnosis of lower extremity radicular pain. Neuro-science. 2013. http://dx.doi.org/10.1155/2013/952570. 10.1155/2013/952570 PMC4045533 24967311

[18] Govind J (2004). Lumbar radicular pain. Aust Fam Physician.

[19] Haig AJ, Geisser ME, Tong HC (2007). Electromyographic and magnetic resonance imaging to predict lumbar stenosis, low-back pain, and no back symptoms. Am J Bone Joint Surg.

[20] Chen HB, Wan Q, Xu QF, Chen Y, Bai B (2016). Reducing surgical levels by paraspinal mapping and diffusion tensor imaging techniques in lumbar spinal stenosis. J Orthop Surg Res.

[21] Chhabra A, Farahani SJ, Thawait GK, Wadhawa V, Belzbrg AJ, Carrino JA (2016). Incremental value of magnetic resonance neurography of lumbosacral plexus over non-contributory lumbar spine magnetic resonance imaging in radiculopathy. A prospective study. World J Radiol.

